# Supplementary material for: Zinc shapes the folding landscape of p53 and establishes a pathway for reactivating structurally diverse cancer mutants
Source: eLife. 2020 Dec 2;9:e61487. doi: 10.7554/eLife.61487 (PMC7728444; doi:10.7554/eLife.61487)
Supplement: Figure 3—source data 1. — ΔGapo and KZn were obtained from fits of the data in Figure 3—figure supplement 1 and Figure 3—figure supplement 2 to Equation 3 (errors are SE of the fits). KZn (direct) values were obtained from Tyr fluorescence spectra as in Figure 2A (errors are SD, n = 3). Asterisks denote that KZn could not be determined due to the presence of the extra Cys in the indicated Tyr-to-Cys mutants. Consequently, Y163C, Y205C, and Y220C are listed as stability-class mutants, but we cannot exclude the possibility that they belong to the mixed zinc-binding/stability class. [file elife-61487-fig3-data1.docx]

| **Mutant** | **ΔG_apo_ (kcal mol^-1^)** | **K_Zn_ (M)** | **K_Zn_ (direct) (M)** |
| --- | --- | --- | --- |
| WT | 6.3 ± 0.1 | 7 ± 2 x 10^-15^ | 1.7 ± 0.7 x 10^-15^ |
| **Zinc binding class** | | | |
| C176S | 5.9 ± 0.1 |  | 2.7 ± 0.8 x 10^-10^ |
| R175H | 6.3 ± 0.2 |  | 5.2 ± 1.8 x 10^-11^ |
| C242S | 5.8 ± 0.1 |  | 2.1 ± 0.6 x 10^-12^ |
| L194F | 7.17 ± 0.06 | 5.0 ± 1.9 x 10^-14^ |  |
| R249S | 6.0 ± 0.1 | 3.3 ± 1.8 x 10^-13^ |  |
| H179R | 6.3 ± 0.1 |  | 1.1 ± 0.5 x 10^-13^ |
| P152L | 6.2 ± 0.3 | 5.4 ± 6.6 x 10^-14^ |  |
| R282Q | 5.8 ± 0.2 | 3.3 ± 1.9 x 10^-14^ |  |
| S241F | 5.8 ± 0.2 | 3.1 ± 1.7 x 10^-14^ |  |
| **Stability class** | | | |
| E285K | 1.7 ± 0.1 | 1.0 ± 0.3 x 10^-14^ |  |
| Y234C | 3.3 ± 0.1 |  | 1.9 ± 0.2 x 10^-15^ |
| Y220C | 4.2 ± 0.1 | * |  |
| Y205C | 3.4 ± 0.1 | * |  |
| Y163C | 4.9 ± 0.2 | * |  |
| V272M | 4.30 ± 0.06 | 2.3 ± 0.4 x 10^-15^ |  |
| A138V | 3.33 ± 0.05 | 1.0 ± 0.2 x 10^-14^ |  |
| G245S | 5.3 ± 0.1 | 3.5 ± 1.7 x 10^-14^ | 7.1 ± 2.5 x 10^-15^ |
| **Mixed zinc binding/stability class** | | | |
| V157F | 3.4 ± 0.2 | 9.5 ± 8.5 x 10^-14^ |  |
| R158H | 5.0 ± 0.2 | 5.1 ± 3.6 x 10^-14^ |  |
| M237I | 5.2 ± 0.2 |  | 3.3 ± 1.5 x 10^-13^ |
| **DNA binding class** | | | |
| R248Q | 7.6 ± 0.5 | 6.5 ± 9 x 10^-15^ | 1.0 ± 0.2 x 10^-15^ |
| R273H | 7.3 ± 0.2 | 1.4 ± 0.9 x 10^-14^ |  |
| R280K | 7.89 ± 0.08 | 1.6 ± 0.9 x 10^-14^ |  |
