# Supplementary material for: Zinc shapes the folding landscape of p53 and establishes a pathway for reactivating structurally diverse cancer mutants
Source: eLife. 2020 Dec 2;9:e61487. doi: 10.7554/eLife.61487 (PMC7728444; doi:10.7554/eLife.61487)
Supplement: Figure 4—source data 1. — KDNA values are reported as mean ± SE in units of μM. Values are derived Hill fitting of fluorescence anisotropy binding curves. Each oligonucleotide was separately analyzed by global fitting across all mutants with floating KDNA and a linked Hill parameter, the value of which is indicated by the bracketed numbers in the top row. Replicates (indicated in the first column) consisted of independent experiments performed with the same preparations of purified proteins and oligonucleotides. Each replicate was fitted with its own KDNA, and the results were pooled. [file elife-61487-fig4-data1.docx]

| **DBD variant (n)** | **waf1 5' (2.6)** | **gadd45 (2.0)** | **puma**  **(2.4)** | **type IV collagenase (2.5)** | **p53rfp**  **(2.2)** | **mdm2**  **(2.0)** | **bax**  **(1.2)** | **rgc**  **(1.0)** | **waf1 3'**  **(nd)** | **EGFR**  **(nd)** |
| --- | --- | --- | --- | --- | --- | --- | --- | --- | --- | --- |
| wt (3) | 0.82 ± 0.07 | 1.1 ± 0.2 | 1.8 ± 0.2 | 2.1 ± 0.3 | 5.0 ± 0.4 | 5.5 ± 1.2 | 12 ± 3 | 13 ± 2 | >25 | >25 |
| e285k (3) | 0.24 ± 0.02 | 0.26 ± 0.05 | 0.34 ± 0.10 | 0.31 ± 0.08 | 0.42 ± 0.12 | 0.21 ± 0.05 | 0.59 ± 0.27 | 0.72 ± 0.30 | >25 | >25 |
| v272m (4) | 0.50 ± 0.06 | 0.80 ± 0.11 | 1.0 ± 0.1 | 1.1 ± 0.1 | 2.0 ± 0.4 | 1.8 ± 0.2 | 9.4 ± 1.1 | 7.0 ± 2.1 | >25 | >25 |
| y234c (3) | 0.46 ± 0.09 | 0.75 ± 0.15 | 1.0 ± 0.1 | 1.2 ± 0.1 | 2.4 ± 0.5 | 2.0 ± 0.3 | 13 ± 3 | 11 ± 4 | >25 | >25 |
| r158h (2) | 0.82 ± 0.16 | 0.94 ± 0.17 | 1.7 ± 0.4 | 2.1 ± 0.6 | 4.4 ± 0.9 | 4.8 ± 1.7 | 12 ± 1 | 7.1 ± 1.6 | >25 | >25 |
| y205c (3) | 0.57 ± 0.12 | 0.88 ± 0.12 | 1.2 ± 0.3 | 1.6 ± 0.3 | 4.1 ± 0.8 | 3.4 ± 1.1 | 15 ± 5 | 7.7 ± 2.1 | >25 | >25 |
| r282q (3) | 0.98 ± 0.16 | 1.4 ± 0.2 | 2.0 ± 0.3 | 2.6 ± 0.3 | 4.0 ± 0.5 | 4.0 ± 0.6 | 12 ± 2 | 13 ± 3 | >25 | >25 |
| v157f (3) | 1.7 ± 1.2 | 2.6 ± 1.7 | 4.1 ± 2.8 | 6.4 ± 4.6 | 8.7 ± 4.7 | 9.2 ± 4.2 | 17 ± 4 | 20 ± 5 | >25 | >25 |
| m237i (3) | 0.83 ± 0.15 | 2.0 ± 0.4 | 2.3 ± 0.4 | 3.0 ± 0.6 | 8.1 ± 1.3 | 7.0 ± 3.7 | >25 | >25 | >25 | >25 |
| y220c (2) | 0.73 ± 0.05 | 1.4 ± 0.1 | 2.4 ± 0.1 | 3.0 ± 0.1 | >25 | >25 | >25 | >25 | >25 | >25 |
| g245s^a^ (4) | 14 ± 6 | 14 ± 11 | 14 ± 11 | 15 ± 10 | 14 ± 11 | 14 ± 11 | 15 ± 10 | 16 ± 9 | >25 | >25 |
| l194f (3) | 3.8 ± 0.2 | 5.8 ± 0.3 | 9.9 ± 0.8 | 14 ± 1 | 24 ± 1 | 23 ± 2 | >25 | >25 | >25 | >25 |
| p152l (4) | 0.79 ± 0.05 | 1.4 ± 0.2 | >25 | >25 | >25 | >25 | >25 | 17 ± 5 | >25 | >25 |
| r273h (2) | >25 | >25 | >25 | >25 | >25 | >25 | >25 | >25 | >25 | >25 |
| r280k (2) | >25 | >25 | >25 | >25 | >25 | >25 | >25 | >25 | >25 | >25 |
| r175h (2) | >25 | >25 | >25 | >25 | >25 | >25 | >25 | >25 | >25 | >25 |
| c242s (2) | >25 | >25 | >25 | >25 | >25 | >25 | >25 | >25 | >25 | >25 |
| s241f (2) | >25 | >25 | >25 | >25 | >25 | >25 | >25 | >25 | >25 | >25 |
| h179r (2) | >25 | >25 | >25 | >25 | >25 | >25 | >25 | >25 | >25 | >25 |
| y163c (2) | >25 | >25 | >25 | >25 | >25 | >25 | >25 | >25 | >25 | >25 |
| r249s (2) | >25 | >25 | >25 | >25 | >25 | >25 | >25 | >25 | >25 | >25 |
| r248q (2) | >25 | >25 | >25 | >25 | >25 | >25 | >25 | >25 | >25 | >25 |
| c176s (2) | >25 | >25 | >25 | >25 | >25 | >25 | >25 | >25 | >25 | >25 |
