# Supplementary material for: Zinc shapes the folding landscape of p53 and establishes a pathway for reactivating structurally diverse cancer mutants
Source: eLife. 2020 Dec 2;9:e61487. doi: 10.7554/eLife.61487 (PMC7728444; doi:10.7554/eLife.61487)
Supplement: Supplementary file 1. — (A) Table of DNA sequences used in p53-p53RE binding experiments. (B) Table of oligonucleotides used to generate p53 mutants by site-directed mutagenesis. (C) Table of exact Student’s t-test p-values for p53 refolding in cells, measured by immunofluorescence (Figure 6A). (D) Table of exact Student’s t-test p-values for p53 refolding in cells, measured by immunofluorescence (Figure 7C) [file elife-61487-supp1.docx]

Supplementary File 1

“Zinc shapes the folding landscape of p53 and establishes a pathway for reactivating structurally diverse cancer mutants”

Adam R. Blanden^1^, Xin Yu^1^, Alan J. Blayney, Christopher Demas, Jeung-Hoi Ha, Yue Liu, Tracy Withers, Darren R. Carpizo^*^, Stewart N. Loh^*^

**Supplementary File 1A: Table of DNA sequences used in p53-p53RE binding experiments**

**Supplementary File 1B: Table of oligonucleotides used to generate p53 mutants by site-directed mutagenesis**

**Supplementary File 1C: Table of exact Student’s t-test p-values for p53 refolding in cells, measured by immunofluorescence (Figure 5A)**

**Supplementary File 1D: Table of exact Student’s t-test p-values for p53 refolding in cells, measured by immunofluorescence (Figure 8C)**

| **Supplemental File 1A: Table of DNA sequences used in p53-p53RE binding experiments** | | |
| --- | --- | --- |
| **Name** | **Sequence (5'-3')** | **5' Cy3 label** |
| gadd45 (forward) | gaacatgtctaagcatgctg | + |
| gadd45 (complement) | cagcatgcttagacatgttc | - |
| puma (forward) | ctgcaagtcctgacttgtcc | + |
| puma (complement) | ggacaagtcaggacttgcag | - |
| mdm2 (forward) | ggtcaagttcagacacgtcc | + |
| mdm2 (complement) | ggacgtgtctgaacttgacc | - |
| rgc (forward) | ggacttgcctggccttgcct | + |
| rgc (complement) | aggcaaggccaggcaagtcc | - |
| p53rfp (forward) | agacaggtcctgacaagcag | + |
| p53rfp (complement) | ctgcttgtcaggacctgtct | - |
| waf1 5' (forward) | gaacatgtcccaacatgttg | + |
| waf1 5' (complement) | caacatgttgggacatgttc | - |
| waf1 3' (forward) | gaagaagactgggcatgtct | + |
| waf1 3' (complement) | agacatgcccagtcttcttc | - |
| bax (forward) | agacaagcctgggcgtgggc | + |
| bax (complement) | gcccacgcccaggcttgtct | - |
| IV collagenase (forward) | agacaagcctgaacttgtct | + |
| IV collagenase (complement) | agacaagttcaggcttgtct | - |
| egfr (forward) | gagctagacgtccgggcagcccc | + |
| egfr (complement) | ggggctgcccggacgtctagctc | - |

| **Supplementary File 1B: Table of oligonucleotides used to generate p53 mutants by site-directed mutagenesis** | | |
| --- | --- | --- |
| **p53 mutation** | **Forward primer (5′–3′)** | **Reverse primer (5′–3′)** |
| Vector | purchased | purchased |
| **Zinc-binding mutants** | | |
| R175H | gttgtgaggcactgcccccac | ctccgtcatgtgctgtgac |
| C176S | gtgaggcgctccccccaccat | aacctccgtcatgtgctgtgac |
| L194F | tcctcagcattttatccgagtggaag | ggggccagaccatcgcta |
| P152L | tccacacccctgcccggcacc | atcaacccacagctgcacagggc |
| R282Q | gggagagaccagcgcacagag | aggacaggcacaaacacg |
| **Stability mutants** | | |
| Y234A | caccatccacgccaactacatgtgtaac | gtacagtcagagccaacc |
| Y234C | accatccactgcaactacatg | ggtacagtcagagccaac |
| V272M | cagctttgagatgcgtgtttg | ttccgtcccagtagattac |
| E285K | ccggcgcacaaaggaagagaa | tctctcccaggacaggcac |
| **Mixed mutant** | | |
| M237I | acaactacatctgtaacagttcct g | agtggatggtggtacagt |
| **DNA-binding mutants** | | |
| R273H | purchased | purchased |
| R280K | tgtcctgggaaagaccggcgc | ggcacaaacacgcacctcaaagc |

| **Supplementary File 1C: Table of exact Student’s t-test p-values for p53 refolding in cells, measured by immunofluorescence (Figure 5A)** | | |
| --- | --- | --- |
| **Mutant Class** | **Mutant** | **p value – ctrl vs ZMC1** |
| Null | TOV112D | <0.0001 |
| Zinc binding | R175H | 0.0001 |
|  | C176S | <0.0001 |
|  | L194F | <0.0001 |
|  | P152L | 0.65 |
|  | R282Q | 0.53 |
| Stability | Y234A | <0.0001 |
|  | Y234C | 0.51 |
|  | V272< | <0.0001 |
|  | E285K | 0.29 |
| Mixed | M237I | <0.0001 |
| DNA binding | R273H | 0.088 |
|  | R280K | 0.2 |

| **Supplementary File 1D: Table of exact Student’s t-test p-values for p53 refolding in cells, measured by immunofluorescence (Figure 8C)** | | |
| --- | --- | --- |
| **Mutant** | **Temperature** | **p value – ctrl vs ZMC1** |
| E285K | 37°C | 0.17 |
|  | 22°C | <0.0001 |
| R175H | 37°C | <0.0001 |
|  | 22°C | 0.0068 |
| TOV112D | 37°C | <0.0001 |
|  | 22°C | 0.0003 |
