# Supplementary material for: Zinc shapes the folding landscape of p53 and establishes a pathway for reactivating structurally diverse cancer mutants
Source: eLife. 2020 Dec 2;9:e61487. doi: 10.7554/eLife.61487 (PMC7728444; doi:10.7554/eLife.61487)
Supplement: Supplementary file 2. [file elife-61487-supp2.docx]

Supplementary File 2

“Zinc shapes the folding landscape of p53 and establishes a pathway for reactivating structurally diverse cancer mutants”

Adam R. Blanden^1^, Xin Yu^1^, Alan J. Blayney, Christopher Demas, Jeung-Hoi Ha, Yue Liu, Tracy Withers, Darren R. Carpizo^*^, Stewart N. Loh^*^

**Supplementary File 2A: Materials and methods for *Mycoplasma* testing**

**Supplementary File 2B: Results of *Mycoplasma* testing**

**Supplementary File 2A: Materials and methods for *Myocoplasma* testing**

In this study we used 11 human tumor cell lines which were purchased from American Type Culture Collection (ATCC; Manassas, VA) and confirmed as mycoplasma free. To perform the mycoplasma test, the cells were seeded in 6-well plates and grown to 50-70% confluency. Cells were harvested and lysed following the instructions provided in the Universal Mycoplasma Detection Kit (ATCC, identifier 30-1012K). PCR reactions were set up using the instructions and reagents contained in the Universal Mycoplasma Detection Kit. The positive control was provided by the kit. The negative control was the PCR master mix with lysis buffer. The PCR product was detected by running the products on a 3 % agarose gel in Tris-acetate-EDTA buffer.

**Supplementary File 2A: Results of *Myocoplasma* testing**

The figure shows the PCR products from *Mycoplasma* tests of the 11 cell lines used in the study, run on a 3 % agarose gel. The presence of *Mycoplasma* is determined by PCR products ranging in size from 434-468 bp (positive control). No such band was detected in any of the cell lines, verifying that they are *Mycoplasma* free.
